# Implicit and explicit attitudes towards disease-modifying antirheumatic drugs as possible target for improving medication adherence

**DOI:** 10.1371/journal.pone.0221290

**Published:** 2019-08-30

**Authors:** M. van Heuckelum, A. J. Linn, L. Vandeberg, R. C. F. Hebing, L. van Dijk, M. Vervloet, M. Flendrie, M. T. Nurmohamed, S. van Dulmen, B. J. F. van den Bemt, C. H. M. van den Ende

**Affiliations:** 1 Department of Rheumatology, Sint Maartenskliniek, Nijmegen, The Netherlands; 2 Department of Pharmacy, Sint Maartenskliniek, Nijmegen, The Netherlands; 3 Amsterdam School of Communication Research, University of Amsterdam, Amsterdam, The Netherlands; 4 Centre for Language Studies, Radboud University, Nijmegen, The Netherlands; 5 Department of Rheumatology, Amsterdam Rheumatology and Immunology Center, Reade, Amsterdam, The Netherlands; 6 Nivel (Netherlands Institute for Health Services Research), Utrecht, The Netherlands; 7 Department of PharmacoTherapy, -Epidemiology, & -Economics (PTEE), Groningen Research Institute of Pharmacy, Faculty of Mathematics and Natural Sciences, University of Groningen, Groningen, The Netherlands; 8 Department of Rheumatology, Amsterdam Rheumatology and Immunology Center, VU University Medical Center, Amsterdam, The Netherlands; 9 Radboud University Medical Center, Radboud Institute for Health Sciences, Department of Primary and Community Care, Nijmegen, The Netherlands; 10 Faculty of Health and Social Sciences, University of South-Eastern Norway, Drammen, Norway; 11 Department of Pharmacy, Radboud University Medical Center, Nijmegen, The Netherlands; 12 Department of Clinical Pharmacy and Toxicology, Maastricht University Medical Centre+, Maastricht, The Netherlands; 13 Department of Rheumatology, Radboud University Medical Center, Nijmegen, The Netherlands; University of Wisconsin Madison School of Pharmacy, UNITED STATES

## Abstract

**Objective:**

This study aims to explore the contribution of implicit attitudes and associations towards conventional disease-modifying antirheumatic drugs (cDMARDs), alongside explicit measures, on medication-taking behaviour and clinical outcomes in adult patients with rheumatoid arthritis (RA).

**Methods:**

In this observational study, implicit attitudes (positive-negative) and health-related associations (health-sickness) were measured with Single Category Implicit Association Tests, whereas explicit outcomes were measured with a bipolar evaluative adjective scale and the Beliefs about Medicines Questionnaire Specific. The primary outcome of this study was medication-taking behaviour subjectively measured by self-report (i.e. validated Compliance Questionnaire on Rheumatology) and objectively measured with electronic drug monitors over a 3 month period. Spearman rank correlations were used to describe correlations between implicit and explicit outcomes. Nested linear regression models were used to assess the additional value of implicit measures over explicit measures and patient-, clinical-, and treatment-related characteristics.

**Results:**

Of the 1659 initially-invited patients, 254 patients with RA agreed to participate in this study. Implicit attitudes correlated significantly with necessity-concerns differential (NCD) scores (ρ *=* 0.13, P = 0.05) and disease activity scores (ρ = -0.17, P = 0.04), whereas implicit health-related associations correlated significantly with mean scores for explicitly reported health-related associations (ρ = 0.18, P = 0.004). Significant differences in age, number of DMARDs, biologic DMARD use, NCD-scores, and self-reported correct dosing were found between the four attitudinal profiles. Nested linear regression models revealed no additional value of implicit measures in explaining self-reported medication-taking behaviour and clinical outcomes, over and above all other variables.

**Conclusion:**

Implicit attitudes and associations had no additional value in explaining medication-taking behaviour and clinical outcomes over and above often used explicitly measured characteristics, attitudes and outcomes in the studied population. Only age and NCD scores contributed significantly when the dependent variable was correct dosing measured with self-report.

## Introduction

Rheumatoid arthritis (RA) is a chronic inflammatory disease characterised by synovial inflammation, which can lead to irreversible articular damage, a decrease in physical functioning and quality of life, and eventually increased healthcare expenditures [1–5]. Conventional disease-modifying antirheumatic drugs (cDMARDs) are the cornerstone of RA treatment and are fundamental to prevent radiologic progression on long-term [1]. Maximum treatment benefits can, however, only be achieved if patients adhere to their treatment [6]. Previous research in patients with RA revealed that adherence rates to DMARDs varied from 30% to 107%, depending on the used measurement method [7]. So far, interventions designed to improve medication adherence were only partly effective in changing medication-taking behaviour [8–10].

An explanation for the ineffectiveness of adherence-improving interventions might be that previous studies have largely focused on patient’s explicit, ‘conscious’, evaluations of e.g. medication or medication-taking behaviour [11,12]. These interventions are often designed on the basis of theories such as the theory of planned behaviour and the health belief model, which form the backbone for understanding how explicit evaluations affect behaviour [11–14]. However, extensive theoretical and empirical contributions in the field of psychology demonstrate that behaviour is only partly driven by conscious, explicit evaluations [11,15–20]. A lot of behaviour originates from more subconscious or automatic processes (i.e. implicit associations) [11,15–20]. Dual process theories, which account for both reflective (conscious) as well as automatic (subconscious) drivers of behaviour, are well-accepted in various scientific domains aimed at unravelling the mechanisms underlying human behaviour (i.e., psychology, behavioural economics, marketing, communication)[21,22]. Adherence research, in contrast, has rarely tapped into this knowledge base, generally ignoring patients’ automatic associations with their medication or medication-taking behaviour. The way to tap into these automatic processes is to infer people’s associations from speeded response tasks (implicit measurement), rather than ask them to introspect (explicit measurement)[23,24]. In this study, we used such implicit measurements with the aim to tap into automatically activated associations that are based on a past experience and mediate favourable or unfavourable feelings that individuals may not be aware of [16,18,21]. We refer to these associations as implicit attitudes, following the evidence that implicit measurements are capable of exposing automatic positive or negative associations, but acknowledging that they do not by definition do so and that implicit refers to the measurement rather than to the process per se [23,25,26]. Explicit attitudes, on the contrary, are defined as deliberate or (sub)conscious evaluations of medication [16,18,21].

To date, few studies have investigated implicit attitudes towards medication and their potential relation to medication-taking behaviour [27–29]. No strong correlation between implicit and explicit attitudes towards medication was previously found, nor between implicit attitudes and self-reported (thus explicitly measured) medication-taking behaviour [27,28]. This was no surprise, given that 1) implicit and explicit processes are known to deviate (cf. dual process models and findings), and 2) self-report to assess medication-taking behaviour is susceptible to the limits of patient’s self-knowledge and the tendency to provide socially desirable answers, and may therefore deviate from actual behaviour [21,30]. Assessing medication-taking behaviour based on self-reports is therefore an important limitation in previous research on implicit and explicit attitudes [27,28]. It can be argued that explicit determinants assessed with self-report correlate more strongly with self-reported behaviour (because both are assumed to be driven by elaborative thought), whereas implicit attitudes may have the potential to correlate uniquely with actual behaviour, which is known to be driven largely by automatic or subconscious processes [23,27]. Objective measurement methods, such as electronic drug monitors, are therefore more suitable to examine implicit attitudes’ relation with actual medication-taking behaviour.

Therefore, the primary objective of this study is to explore the contribution of implicitly measured attitudes towards cDMARDs, alongside explicit measures, on medication-taking behaviour (i.e. measured subjectively with self-report and objectively with electronic drug monitors) of patients with RA. The secondary objective is to explore the contribution of implicitly measured attitudes towards cDMARDs, alongside explicit measures, on disease activity scores in patients with RA.

## Methods

### Study design and setting

An observational study was conducted in two of the largest specialised rheumatology centres across the Netherlands (i.e. covering approximately 20% of all patients with RA): Sint Maartenskliniek (Nijmegen) and Reade (Amsterdam). Participants were recruited between July 5^th^, 2016 and November 30^th^, 2017. The STROBE (Strengthening the Reporting of Observational Studies in Epidemiology) statement for observational studies and EMERGE (ESPACOMP Medication Adherence Reporting Guideline) were used as guidance for adequate reporting of this study (See S1 Table for all the abbreviations used in this study) [31,32].

### Eligibility criteria and patient selection

Consecutive adult (≥ 18 years) patients with a clinical diagnosis of RA, treated with at least one cDMARD for a minimum period of one year were invited to participate in this study. No additional in- and exclusion criteria were defined. Four weeks before their planned regular consultation with their treating clinician, patients received written information and an informed consent form. After one to two weeks, patients were approached by telephone to ask about their willingness to participate in this study. In case patients agreed to participate, the researcher planned a research appointment before their regular consultation in order to sign the informed consent form and complete baseline measurements.

### Study outcomes

Primary outcome of this study was medication-taking behaviour measured over a period of three months after inclusion. Both self-report (i.e. the validated Compliance Questionnaire on Rheumatology (CQR)) and electronic drug monitors (i.e. Medication wAardex®)) were used to assess the implementation of the dosing regimen [33–35]. The operational definition for the implementation of dosing regimens was correct dosing, which is defined as the proportion of days with the correct number of doses taken [33,36]. Patients were considered to be adherent if the proportion of correct days was >80% based on the prescribed medication regimen by the health professional. Continuous adherence data were used for nested linear regression models. Patient’s disease activity score (DAS28-CRP) was the secondary outcome measure, which was assessed in conformity with treatment protocols as part of standard care [1,37].

### Variables and data collection

Baseline measurements were performed in the consulting room of the outpatient pharmacy. The following data were collected with a hardcopy questionnaire at baseline: socio-demographic characteristics (i.e. age, sex, educational level, living status, ethnicity), explicit attitudes and health-related associations with cDMARDs with a bipolar evaluative adjective scale (8 items to evaluate positive-negative attitudes, 10 items to evaluate health-sickness related associations), beliefs about medicines with the Beliefs about Medicines Questionnaire Specific (BMQ-Specific, 10 Likert-scaled items, ranging from 1 to 5), and self-reported medication-taking behaviour with the validated CQR with 19 Likert-scaled (ranging from 1 to 4) items [33,34,38]. Implicit attitudes and health-related associations were collected with Single Category Implicit Association Tests (SC-IATs), which were performed on a laptop in the consulting room of the outpatient pharmacy [24,39,40]. Clinical characteristics (i.e. disease duration, serology, type and current number of DMARD(s), disease activity scores) were extracted from patient’s medical file by the local researchers. Patient diaries were handed at baseline in order to let patients register possible unintended openings of MEMS. Follow-up measurements were performed at the day of the next planned regular consultation, with a minimum and maximum interval between baseline- and follow-up visits of three and nine months, respectively. At follow-up visit, MEMS read-outs were used to assess correct dosing over the previous months.

### Measurement instruments

#### Single category implicit association tests (SC-IATs)

SC-IATs (Inquisit version 5) were used to measure automatic associations [24,39–41]. The SC-IAT is considered a reliable and valid instrument to measure implicit associations, which in this study is constituted of two concepts, i.e. attitudes towards cDMARDs (positive versus negative) and health-related associations with cDMARDs (health versus sickness). The measurement of each concept included three rounds: one practice round of 20 trials followed by two experimental rounds of 40 trials each (see S1 File). Each trial was defined as a computerised categorisation task in which automatic associations were measured based on patient’s response times. The response times in the experimental rounds serve as a proxy for association strength, where faster responses represent stronger associations. For instance, if patients were on average faster in trials coupling drug stimuli and positive (versus negative) stimuli, this reflects a relatively positive (versus negative) automatic association with cDMARDs. S1 File provides a more detailed description of the SC-IATs procedures used in this study. The design and procedures of the SC-IATs were based on the pilot study of Linn *et al* [27].

#### Explicit medication attitudes, health-related associations and beliefs about medicines

Explicit attitudes (10 items, e.g. I think [name cDMARD] is 1 negative– 5 positive) and explicit health-related associations (8 items, e.g. ‘to what extent do you associate [name cDMARD] with the following terms’, 1 dead– 5 alive) were assessed with a bipolar evaluative adjective scale. The items were identical to those in the SC-IATs (see S2 File for the complete list of words used in the bipolar evaluative adjective scale). Medication necessity and concern beliefs were assessed with the validated Beliefs about Medicines Questionnaire (BMQ) Specific (10 items, 5 necessity items and 5 concern items) [38]. Item scores varied from 1 (strongly disagree) to 5 (strongly agree), which resulted in sum scale scores of 5 to 25 for each subscale (necessity beliefs versus concern beliefs).

### Study size

A common rule of thumb is to formulate sample size requirements as events per variable, with a minimum of 10 events per variable. Assuming a sample size requirement of 10 non-adherent patients per variable and a prevalence of 33% of non-adherence, a sample of 240 patients is sufficient to build a reliable logistic model including a maximum of 8 independent variables. Taking into account 15% loss to follow-up, a sample size of 275 patients was required.

### Statistical methods

Statistical analyses were performed with STATA version 13.1. Descriptive statistics were used to describe patient characteristics by using mean (SD) or median (P25-P75) depending on the distribution of measurements. Educational level was classified in low, moderate or high educational level. Low educational level was defined as no education, (extended) primary education or pre-vocational education, moderate educational level was defined as vocational education or selective secondary education, and high educational level was defined as education provided by universities of applied sciences and research universities. Data were presented as percentages in case of proportions. P-values ≤0.05 were considered statistically significant.

Implicit attitudes and health-related associations were assessed in terms of response times in milliseconds (ms) on the SC-IATs. The improved IAT scoring algorithm described by Greenwald *et al*. was used as scoring procedure to calculate the *D* measure for strength of automatic associations [42]. S1 File provides a detailed description of the statistical methods to calculate *D* measures. *D* measures above 0 indicated that patients had relatively faster response times on positive categorisation rounds than on negative categorisation rounds, and were interpreted as a relatively more positive than negative implicit attitude towards cDMARDs or a relatively more health-related association than sickness-related association, and vice versa.

For explicit attitudes, mean sum scale scores were calculated for each concept (i.e. attitudes and health-related associations). Patients with a mean scale score below or equal to 3 were considered to be relatively negative or have sickness-related associations with cDMARDs, whereas patients with mean scale scores higher than 3 were considered to be relatively positive or associated their cDMARD use with health. Beliefs about medicines were operationalised as necessity-concerns differential (NCD) scores [38,43]. This NCD was calculated by subtracting the sum of the item scores for concerns from the sum of item scores for necessity beliefs. A negative NCD indicated that concern beliefs predominate necessity beliefs and vice versa [38,43].

Participants were also categorised in attitudinal profiles based on their (in)congruent (implicit and explicit) attitudes and health-related associations based on the cut-off scores for implicit and explicit attitudes and health-related associations as described above. Depending on the distribution and type of variables, Two-sample *t*-tests, Pearson chi-square tests, Fisher’s exact tests, Bonferroni corrected post-hoc tests, and proportion tests were performed to test for significant differences in patient characteristics between study sites and attitudinal profiles.

Self-reported medication-taking behaviour was calculated with the discriminant function for CQR items as described by de Klerk *et al* [33]. The critical cut-off score of -2.0046 for correct dosing ≤80% was used to identify adherent and non-adherent patients [33]. Medication-taking behaviour measured with self-report was compared with medication-taking behaviour measured with MEMS. The percentage of adherent patients per day was calculated over time during three months follow-up and presented per attitudinal profile. The proportion of patients in remission was presented for each attitudinal profile based on the cut-off scores for DAS28-CRP and DAS28-BSE, as described by Fleischmann *et al* [37].

Spearman rank correlations were used to describe the correlation between patient’s implicit and explicit outcomes, medication-taking behaviour (i.e. measured by self-report and using MEMS), and clinical outcomes. Because of the explorative (rather than hypothesis-testing) character of this study, no multiple testing corrections were performed over the separate correlational analyses. Nested linear regression models (i.e. by sequentially adding blocks of variables) were used to assess the additional value of implicit measures, over explicit measures and patient characteristics, in explaining adherence to medication and disease activity scores in patients with RA. For each separate model three dependent continuous variables were used: correct dosing based on (1) the CQR discriminant function, and (2) MEMS data, followed by (3) DAS28-CRP scores at baseline.

### Ethical approval

This study was conducted according to the ethical principles for medical research as stated in the Declaration of Helsinki (64^th^ WMA General Assembly, Fortaleza, Brazil, October 2013) and was approved by the Medical Research Ethics Committee of Arnhem-Nijmegen (File: 2016–2410).

### Patient and public involvement

Two patient research partners were involved in the design phase of this study. Those patient research partners pretested the Single Category Implicit Association Tests and assessed the comprehensibility of the hardcopy questionnaire for patients with rheumatoid arthritis.

## Results

### Study sample characteristics

**Fig 1:**
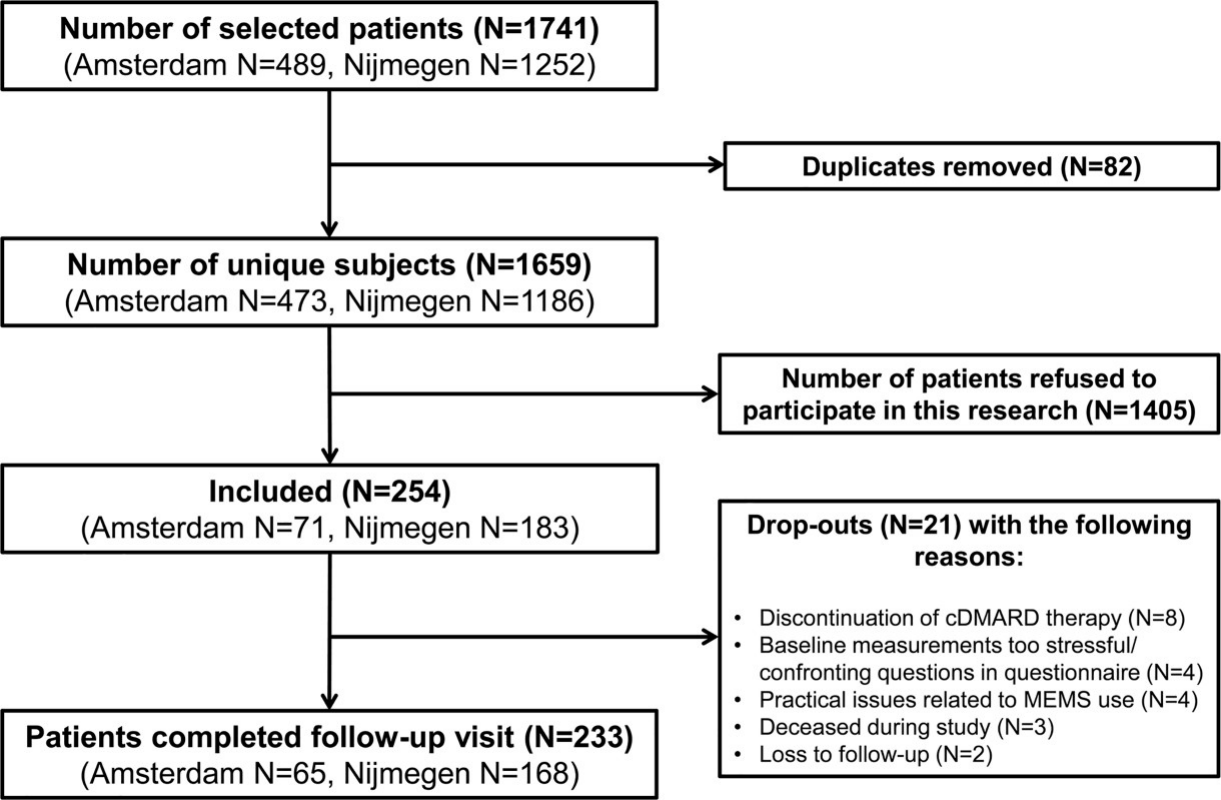
Flowchart of study participants.

Of the 1,659 initially invited patients, 254 patients agreed to participate in this study. The overall response rate was 15.3% (Nijmegen: 15.4%; Amsterdam: 15.0%). Fig 1 presents an overview of patient recruitment, patient inclusion and drop-outs during follow-up. Participants had a mean age of 62.8 (SD:11.2) years, 68.1% was female, 32.9% of the patients was highly educated, and 22.2% was living alone. The mean disease duration of patients was 11.8 (SD:9.0) years and biologic DMARDs were prescribed to 32.7% of the included patients. Methotrexate tablets were significantly more often prescribed in Amsterdam compared to Nijmegen (resp. 77.5% versus 40.4%, P<0.0001), whereas methotrexate subcutaneous injections were significantly more often prescribed in Nijmegen than in Amsterdam (resp. 31.1% versus 7.0%, P = 0.0001). Table 1 provides an overview of all patient characteristics. Due to different treatment protocols applied at both study sites, DAS28-CRP scores were only available for patients treated in Nijmegen (N = 152).

**Table 1:** Study sample characteristics.

Study sample characteristics at baseline		Amsterdam (N = 71)	Nijmegen (N = 183)	Overall (N = 254)	P-value
**Patient characteristics**					
	Age in years, mean (SD)	63.4 (11.2)	62.5 (11.2)	62.8 (11.2)	0.60
	Female, N (%)	49 (69.0)	124 (67.8)	173 (68.1)	0.85
	High educational level, N (%)	26 (36.6)	57 (31.1)	83 (32.7)	0.44
	Living alone, N (%)	**27 (38.0)**	**29 (15.8)**	**56 (22.0)**	**<0.001**
	Dutch ethnic background, N (%)	68 (95.8)	176 (96.2)	244 (96.1)	0.55
**Disease characteristics**					
	Disease duration in years, mean (SD)	10.9 (8.8)	12.1 (9.1)	11.8 (9.0)	0.34
	Rheum factor positive serology, N (%)	48 (67.6)	123 (67.2)	171 (67.3)	0.85
	Anti-CCP positive serology, N (%)	50 (70.4)	116 (63.4)	166 (65.4)	0.44
	Number of comorbidities, mean (SD)	2.0 (1.7)	2.2 (1.8)	2.1 (1.8)	0.45
	Proportion of RA patients in remission, N (%)	**11 (15.5)**	**95 (51.9)**	**106 (41.7)**	**0.04**
**Treatment characteristics**					
	Number of DMARDs, mean (SD)	1.5 (0.7)	1.6 (0.5)	1.6 (0.6)	0.62
	Corticosteroids, N (%)	**32 (45.1)**	**31 (16.9)**	**63 (24.8)**	**<0.001**
	Methotrexate oral, N (%)	**55 (77.5)**	**74 (40.4)**	**129 (50.8)**	**<0.001**
	Methotrexate subcutaneous, N (%)	**5 (7.0)**	**57 (31.1)**	**62 (24.4)**	**<0.001**
	Leflunomide, N (%)	**2 (2.8)**	**22 (12.0)**	**24 (9.4)**	**0.02**
	Sulfasalazine, N (%)	**10 (14.1)**	**11 (6.0)**	**21 (8.3)**	**0.04**
	Azathioprine, N (%)	**0 (0.0)**	**12 (6.6)**	**12 (4.7)**	**0.03**
	Hydroxychloroquine, N (%)	23 (32.4)	40 (21.9)	63 (24.8)	0.08
	Biologic DMARDs, N (%)	**13 (18.3)**	**70 (38.2)**	**83 (32.7)**	**<0.01**
	Other drugs than DMARDs, mean (SD)	**4.8 (3.5)**	**6.9 (4.6)**	**6.3 (4.4)**	**<0.0001**
**Self-reported medication-taking behaviour**					
	CQR correct dosing, adherent, N (%)	62 (87.3)	170 (92.9)	232 (91.3)	0.08

Categories were high versus medium-low for educational level, living alone versus living together (with children and/or partner) for residential status, and for ethnic background Dutch versus other. High educational level included a university degree or a degree in universities of applied sciences. P-values were calculated by Pearson chi-square tests or Two-sample *t*-tests. P-values ≤0.05 were considered statistically significant. Abbreviations: CQR (Compliance Questionnaire on Rheumatology); DMARDs (disease-modifying antirheumatic drugs); RA (rheumatoid arthritis).

Of the 254 patients who agreed to participate, 98.8% completed all SC-IATs, 98.0% the bipolar evaluative adjective scale, 98.8% the BMQ-Specific, 99.2% the CQR and 91.7% of the patients provided MEMS data. The main reason for missing MEMS data was discontinuation of cDMARD therapy (Fig 1). Continuous CQR adherence data instead of dichotomous data were used in this study, since the validation of the CQR against MEMS together with the arbitrary cut-off score of 80% has been subject to considerable debate [44,45]. The proportion of correct trials in the SC-IAT experimental rounds for the positive-negative concept was 93.3% and for the health-sickness concept 95.5%. No participants were excluded for further data-analysis of implicit data.

### Patient’s implicit and explicit attitudes and associations, including beliefs about medication

The mean *D* measure for implicit attitudes towards cDMARDs was -0.054 (SD: 0.42, range: -1.26; 1.21, skewness: -0.11, kurtosis: 2.81), whereas the mean *D* measure for implicit health-related associations with cDMARDs was -0.10 (SD: 0.38, range: -1.25; 1.03, skewness: -0.03, kurtosis: 3.24). The mean score for explicit attitudes of patients who completed the bipolar evaluative adjective scale was 3.5 (SD:0.72, range: 1.4; 5, skewness: -0.24, kurtosis: 3.25), which was similar for explicit health-sickness associations with cDMARDs (*M* = 3.6, SD: 0.89, range: 1; 5, skewness: -0.26, kurtosis: 2.47). Regarding beliefs about medicines, the mean sum scale score for necessity beliefs and for concern beliefs was 19.9 (SD: 3.6, range: 5; 25, skewness: -0.78, kurtosis: 3.9) and 14.1 (SD: 3.9, range: 5; 25, skewness: -0.18, kurtosis: 2.58), respectively. The mean necessity-concerns differential score of 5.8 (SD: 5.2, range: -16; 19, skewness: -0.17, kurtosis: 3.8) indicates that necessity beliefs outweigh patient’s concerns about medication.

**Fig 2:**
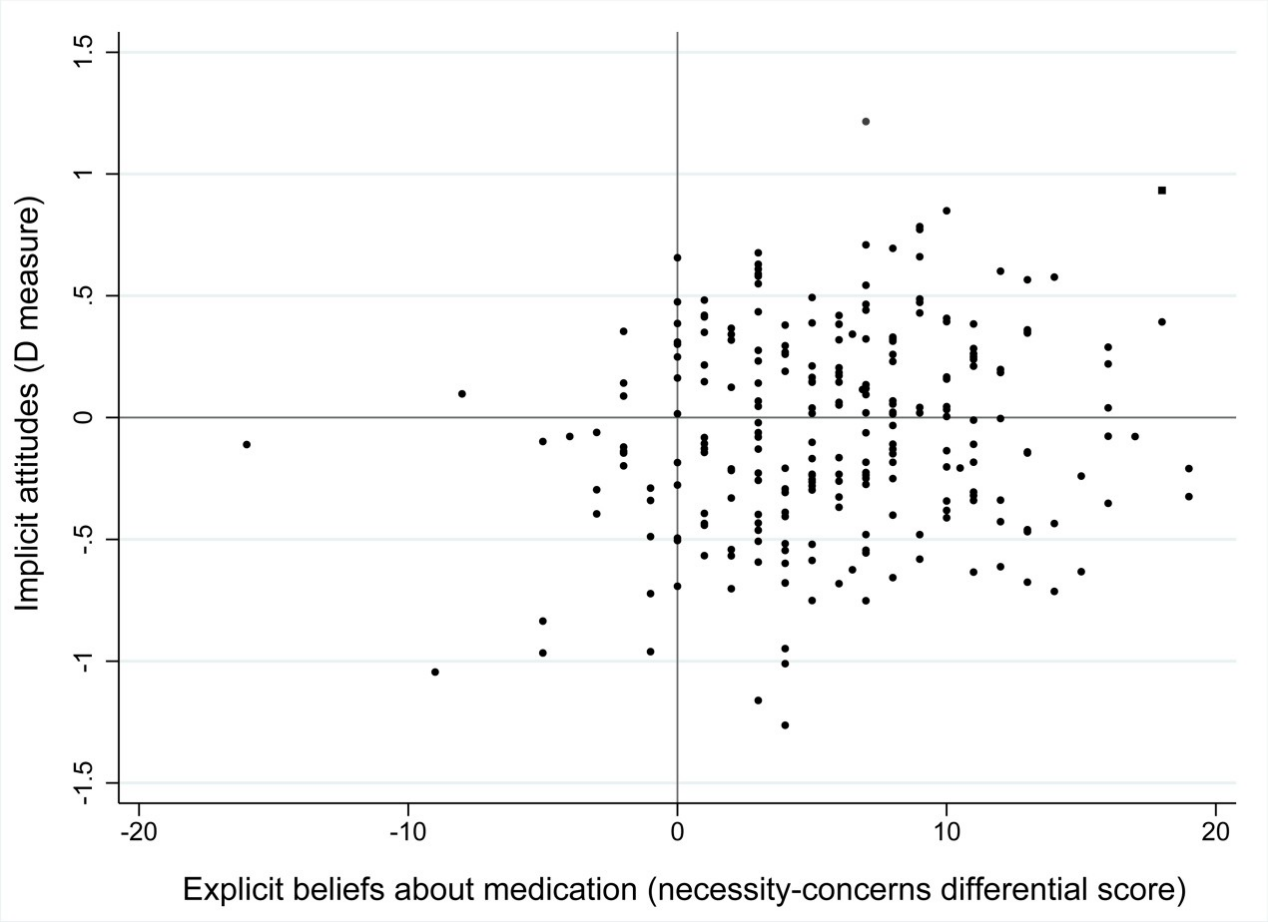
Correlation between implicit attitudes (*D* measure) and explicit beliefs about medication (necessity-concerns differential scores). Higher NCD-scores indicate that necessity beliefs outweigh concern beliefs, whereas higher *D* measures for implicit attitudes indicate more positive attitudes towards cDMARDs (ρ = 0.13, P = 0.05). Abbreviations: cDMARD (conventional disease-modifying antirheumatic drug), NCD (necessity-concerns differential).

Patient’s implicit attitudes (*D* measure) and explicit attitudes (mean score measured with the bipolar evaluative adjective scale) were not significantly correlated (ρ = 0.09, P = 0.16), however a significant weak correlation was found between mean *D* measures for implicit attitudes and necessity-concerns differential scores (ρ *=* 0.13, P = 0.05, illustrated in Fig 2). Also implicit health-related associations were significantly (yet weakly) correlated with mean scores for explicitly reported health-related associations (ρ = 0.18, P = 0.004). No significant correlation was found between implicit health-related associations and NCD-scores (ρ = 0.09, P = 0.18).

Implicit attitudes (*D* measure) were not correlated with self-reported correct dosing (ρ = -0.03, P = 0.68), nor with MEMS correct dosing (ρ = 0.06, P = 0.40). However, a weak significant negative correlation between *D* measures for implicit attitudes and disease activity scores was found (ρ = -0.17, P = 0.04), where more negative implicit attitudes (*D* measures) were associated with higher disease activity scores. No correlations were found between implicit health-related associations and any of the following variables: correct dosing measured with self-report (ρ = 0.04, P = 0.51), correct dosing measured with MEMS (ρ = -0.002, P = 0.97), and disease activity scores (ρ = -0.07, P = 0.42).

Explicit attitudes (measured with a bipolar evaluative adjective scale) were not correlated with self-reported correct dosing (ρ = 0.10, P = 0.12), MEMS correct dosing (ρ = 0.10, P = 0.13), and disease activity scores (ρ = -0.09, P = 0.30). Also explicit health-related associations were not correlated with MEMS correct dosing (ρ = 0.11, P = 0.08), however, a significant negative correlation was found between explicit health-related associations and disease-activity scores (ρ = -0.21, P = 0.01), and a significant positive correlation with self-reported correct dosing (ρ = 0.13, P = 0.05). NCD-scores were significantly correlated with self-reported correct dosing (ρ = 0.26, P<0.0001), but not with MEMS correct dosing and disease activity scores.

### Congruent and incongruent (implicit and explicit) attitudinal profiles

Fewer patients displayed implicit positive attitudes (47.2%) and health-related associations (39.6%) regarding cDMARD therapy than when explicitly asked (75.6% and 67.3%, respectively). Based on *D* measures for implicit attitudes and mean scores for explicit attitudes from the bipolar evaluative adjective scale, patients were categorized in four attitudinal profiles per concept, indicating the (in)congruently positive or negative nature of their implicit and explicit attitudes (see Table 2 for an overview of subgroup characteristics for each attitudinal profile for the concept positive-negative). About half of all participants displayed incongruent (implicit and explicit) attitudes and health-related associations (46.3% and 49.0%, respectively).

**Table 2:** Description of profiles of patients diagnosed with rheumatoid arthritis based on (in)congruent implicit and explicit attitudes.

		Profile I	Profile II	Profile III	Profile IV	P-value
**Attitudes towards cDMARDs**	**Explicit**	Positive	Positive	Negative	Negative	
	**Implicit**	Positive (N = 94)	Negative (N = 92)	Negative (N = 38)	Positive (N = 22)	
**Patient characteristics**						
Age in years, mean (SD)		**63.8 (10.7)**	**63.2 (11.4)**	**57.7 (12.5)**	**62.5 (7.7)**	**0.05**
Female, N (%)		62 (66.0)	60 (65.2)	29 (76.3)	15 (68.2)	0.64
High educational level, N (%)		39 (41.5)	23 (25.0)	11 (28.9)	9 (40.9)	0.09
Living alone, N (%)		22 (23.4)	20 (21.7)	10 (26.3)	2 (9.1)	0.45
**Clinical characteristics**						
Disease duration in years, mean (SD)		10.9 (9.0)	12.2 (8.1)	10.3 (9.0)	15.9 (12.3)	0.10
Anti-CCP positive, N (%)		58 (61.7)	64 (69.6)	23 (60.5)	16 (72.7)	0.49
Number of comorbidities, mean (SD)		**2.3 (1.9)**	**2.3 (1.8)**	**1.5 (1.6)**	**2.0 (1.4)**	**0.04**
**Treatment characteristics**						
Number of DMARDs, mean (SD)		**1.6 (0.6)**	**1.5 (0.6)**	**1.7 (0.6)**	**1.4 (0.7)**	**0.03**
Using bDMARDs, N (%)		**27 (28.7)**	**29 (31.5)**	**21 (55.3)**	**3 (13.6)**	**0.005**
**Beliefs about medicines**						
Necessity-concerns differential, mean (SD)		**6.7 (4.3)**	**6.7 (5.1)**	**1.7 (5.6)**	**4.7 (4.6)**	**0.0001**
**Study outcomes**						
Correct dosing: proportion of adherent patients based on self-report, N (%)		**87 (92.6)**	**87 (94.6)**	**30 (78.9)**	**22 (100)**	**0.01**
Correct dosing, proportion of adherent patients based on MEMS, N (%)		77 (84.6)	76 (90.5)	25 (75.8)	16 (76.2)	0.15
DAS28-CRP, mean (SD)		2.15 (1.0)	2.13 (0.9)	2.6 (1.2)	2.4 (1.5)	0.34
Proportion of patients in remission, N (%)		40 (42.6)	41 (44.6)	13 (34.2)	8 (36.4)	0.47

Categories were low versus medium-high for educational level, living alone versus living together (with children and/or partner) for residential status, and for ethnic background Dutch versus other. Variables with unadjusted P-values ≤0.05 were considered statistically significant and were further analysed with Bonferroni corrected post-hoc tests. Abbreviations: DMARDs (disease-modifying antirheumatic drugs), bDMARD (biologic DMARD), MEMS (Medication Event Monitoring System), DAS28-CRP (Disease Activity Score based on 28 joints and C-Reactive Protein).

**Fig 3:**
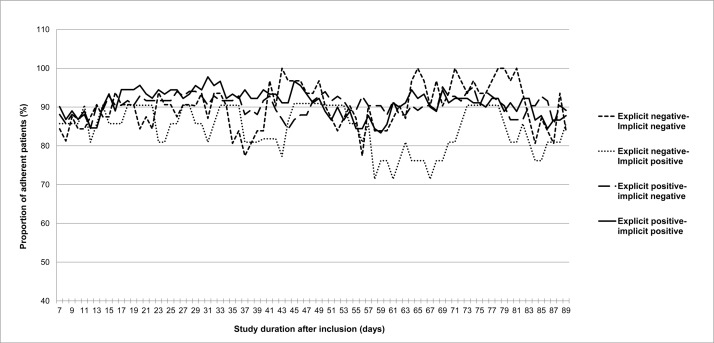
The proportion of adherent patients with rheumatoid arthritis over time based on MEMS correct dosing adherence. Based on *D* measures and mean scale scores for explicit attitudes, patients were categorized in (in)congruent positive or negative attitudinal profiles.

Bonferroni corrected post-hoc tests revealed that congruently negative patients (profile III) differed significantly in age, NCD-scores, bDMARD use and self-reported correct dosing from other attitudinal profiles. Congruently negative patients were on average 6.1 years younger (95%CI: -11.74; -0.46, P = 0.03) than congruently positive patients, reported more concerns than explicitly positive patients (contrast with profile I: -4.92, 95%CI: -7.42; -2.41, P<0.001; contrast with profile II: -4.92, 95%CI: -7.46; -2.43, P<0.001), more often used bDMARDs than all other attitudinal profiles (contrast with profile I: 26.5%, P = 0.004; contrast with profile II: 23.7%, P = 0.01; contrast with profile IV: 41.6%, P = 0.001) and were less often adherent based on self-reported correct dosing than the other attitudinal profiles (contrast with profile I: 13.6%, P = 0.03; contrast with profile II: 15.6%, P = 0.007; contrast with profile IV: 21.1%, P = 0.02). When exploring the proportion of adherent patients measured with MEMS over time between different profiles, similar patterns emerge within the four attitudinal profiles (Fig 3). However, the proportion of adherent patients with negative explicit attitudes (profiles III and IV) more often dropped below the 80% level than for the positive explicit attitudinal profiles (I and II). The small number of patients categorised in these explicitly negative profiles might have contributed to these findings. Additionally, the largest difference in proportion of adherent patients between both measurement instruments (self-report versus MEMS) was found for patients assigned to profile IV. No significant differences between attitudinal profiles were found in the number of comorbidities and the total number of DMARDs after Bonferroni adjusted post-hoc tests.

Subgroup characteristics for each profile regarding health-related associations can be found in S2 Table. Patients who displayed congruently sickness-related associations with cDMARDs differed significantly in NCD-scores from patients who reported explicit (regardless of their implicit) health-related associations (contrast with profile I: -4.1, P<0.001; contrast with profile II: -3.0, P = 0.002). In other words, patients who displayed congruent (implicit and explicit) sickness-related associations with cDMARDs had more concerns about their medication than patients who reported explicit health-related associations with cDMARDs. The same applied for patients categorised in profile IV compared with patients categorised in profile I (contrast: -6.2, P<0.001) and profile II (contrast:-5.0, P<0.001). The mean number of DMARDs in the congruently sickness-related subgroup was significantly higher than the mean number of DMARDs in the congruently health-related subgroup (contrast: 0.29, P = 0.04). When exploring the proportion of adherent patients measured with MEMS over time between the different health-related attitudinal profiles, results were similar to those of the positive-negative concept (data not shown).

### Added value of implicit attitudes in explaining medication-taking behaviour and clinical outcomes

The skewed MEMS data (i.e. high proportion of adherent patients), even after data transformation, did not meet the assumptions for linear regression modelling. Medication-taking behaviour measured with MEMS was, therefore, excluded for nested linear regression modelling. Nested linear regression models showed that only age and NCD-scores contributed significantly when the dependent variable was correct dosing measured with self-report. However, no variables measured in this study contributed significantly to disease activity scores. Implicit attitudes and implicit health-related associations, alongside patient characteristics and explicit measures, had no added value in explaining variance in medication-taking behaviour when measured with self-report. The same applied for disease activity scores (see Table 3 and Table 4). Overall, determinants measured in this study explained approximately 11 to 17% of the variance in medication-taking behaviour and disease activity scores.

**Table 3:** Results nested linear regression models: Contribution of implicit attitudes over and above patient characteristics, disease characteristics, treatment characteristics and explicit measures in patients with rheumatoid arthritis.

	Correct dosing: self-report		DAS28-CRP	
	*Coefficient*	*R*^*2*^	*Coefficient*	*R*^*2*^
	*(95%CI)*	*(ΔR*^*2*^*)*	*(95%CI)*	*(ΔR*^*2*^*)*
**Block 1: Patient characteristics**				
Age (years)	0.02*		-0.01	
	(0.003; 0.04)		(-0.03; 0.01)	
Female (y/n)	-0.40		0.27	
	(-0.82; 0.02)		(-0.13; 0.67)	
High educational level	0.20		-0.20	
	(-0.21; 0.60)		(-0.60; 0.20)	
Living alone (y/n)	-0.11		0.17	
	(-0.61; 0.39)		(-0.35; 0.69)	
Hospital Nijmegen (y/n)	0.07		NA	
	(-0.39; 0.52)			
		**0.064**		**0.044**
**Block 2: Disease characteristics**				
Disease duration (years)	-0.01		-0.002	
	(-0.03; 0.01)		(-0.023; 0.02)	
Anti-CCP positive (y/n)	-0.06		0.03	
	(-0.50; 0.37)		(-0.38; 0.45)	
Number of comorbidities	0.02		-0.03	
	(-0.11; 0.16)		(-0.16; 0.09)	
		**0.076**		**0.046**
		**(0.012)**		**(0.002)**
**Block 3: Treatment characteristics**				
Number of DMARDs	0.06		-0.07	
	(-0.34; 0.47)		(-0.57; 0.44)	
bDMARD use (y/n)	0.10		0.15	
	(-0.44; 0.64)		(-0.42; 0.71)	
N of other drugs than DMARDs	0.04		0.05	
	(-0.02; 0.10)		(-0.002; 0.096)	
		**0.098**		**0.075**
		**(0.022)**		**(0.028)**
**Block 4: Beliefs about medicines**				
Necessity-concerns differential score	0.08**		-0.02	
	(0.04; 0.13)		(-0.06; 0.02)	
		**0.163**		**0.097**
		**(0.065)**		**(0.023)**
**Block 5: Explicit attitudes (bipolar evaluative adjective scale): positive versus negative**				
Mean score for explicit attitudes	-0.09		-0.16	
	(-0.40; 0.22)		(-0.46; 0.13)	
		**0.165**		**0.107**
		**(0.002)**		**(0.009)**
**Block 6: Implicit attitudes (concept: positive versus negative)**				
*D* measure	0.01		-0.007	
	(-0.45; 0.48)		(-0.43; 0.41)	
**Total R**^**2**^		**0.165**		**0.107**
		**(0.000)**		**(0.000)**

Level of significance

* P-value<0.05

**P-value<0.001

NA: Not applicable

**Table 4:** Results nested linear regression models: contribution of implicit health-related associations over and above patient characteristics, disease characteristics, treatment characteristics and explicit measures in patients with rheumatoid arthritis.

	Correct dosing: self-report		DAS28-CRP	
	*Coefficient*	*R*^*2*^	*Coefficient*	*R*^*2*^
	*(95%CI)*	(Δ*R*^*2*^)	*(95%CI)*	(Δ*R*^*2*^)
**Block 1: Patient characteristics**				
Age (years)	0.02*		-0.009	
	(0.003; 0.04)		(-0.03; 0.011)	
Female (y/n)	-0.40		0.25	
	(-0.82; 0.02)		(-0.15; 0.64)	
High educational level	0.20		-0.20	
	(-0.20; 0.61)		(-0.59; 0.19)	
Living alone (y/n)	-0.11		0.15	
	(-0.62; 0.39)		(-0.37; 0.67)	
Hospital Nijmegen (y/n)	0.06		NA	
	(-0.39; 0.51)			
		**0.064**		**0.044**
**Block 2: Disease characteristics**				
Disease duration (years)	-0.01		-0.001	
	(-0.03; 0.01)		(-0.22; 0.02)	
Anti-CCP positive (y/n)	-0.06		0.06	
	(-0.49; 0.37)		(-0.36; 0.47)	
Number of comorbidities	0.02		-0.04	
	(-0.12; 0.16)		(-0.17; 0.08)	
		**0.076**		**0.046**
		**(0.012)**		**(0.002)**
**Block 3: Treatment characteristics**				
Number of DMARDs	0.06		-0.10	
	(-0.35; 0.47)		(-0.61; 0.41)	
bDMARD use (y/n)	0.11		0.14	
	(-0.43; 0.64)		(-0.43; 0.70)	
N of other drugs than DMARDs	0.04		0.05	
	(-0.02; 0.10)		(-0.003; 0.09)	
		**0.098**		**0.075**
		**(0.022)**		**(0.029)**
**Block 4: Beliefs about medicines**				
Necessity-concerns differential score	0.08**		-0.02	
	(0.04; 0.12)		(-0.05; 0.02)	
		**0.163**		**0.098**
		**(0.065)**		**(0.023)**
**Block 5: Explicit associations (bipolar evaluative adjective scale): health versus sickness**				
Mean score for explicit associations	-0.03		-0.19	
	(-0.27; 0.22)		(-0.42; 0.03)	
		**0.164**		**0.119**
		**(0.001)**		**(0.021)**
**Block 6: Implicit associations (concept: health versus sickness)**				
*D* measure	-0.005		-0.03	
	(-0.53; 0.52)		(-0.54; 0.48)	
**Total R**^**2**^		**0.164**		**0.119**
		**(0.000)**		**(0.000)**

Level of significance

* P-value<0.05

**P-value<0.001

NA: Not applicable

## Discussion

This study showed that implicit attitudes and health-related associations do not explain medication-taking behaviour and clinical outcomes in clinical practice over and above 1) patient characteristics, 2) clinical variables, 3) treatment characteristics, 4) explicit attitudes and health-related associations, or 5) beliefs about medicines. Only age and NCD-scores contributed significantly to medication taking behaviour measured with self-report. However, some significant but weak associations were found between implicit attitudes and necessity-concerns differential scores, between implicit and explicit health-related associations, and implicit attitudes and disease activity. The categorization of patients in combined implicit and explicit attitudinal profiles revealed that characteristics of in particular patients with congruently negative or congruently sickness-related associations, differed from other attitudinal profiles.

To our knowledge, this is the first study which compares groups of patients with congruent and incongruent implicit and explicit attitudes towards medication in relation to objectively measured medication-taking behaviour and clinical outcomes in patients with RA. Linn *et al* already emphasized the importance of exploring implicit associations as possible targets for improving actual (rather than self-reported) medication adherence in this population [27]. In terms of (in)congruent implicit and explicit attitudes towards medication, our findings are in line with the findings reported by Linn *et al* and Rüsch *et al*, which supports the idea that implicit and explicit measures are different but related constructs [27,28]. However, contrary to our expectations, patient’s implicit associations did not significantly explain variance in objectively measured medication-taking behaviour. Several possible explanations can be given to elucidate this finding.

One explanation is that no association between implicit associations and objectively measured medication-taking behaviour in clinical practice exist. In other words, implicit associations (with cDMARDs) do not underlie (medication-taking) behaviour. This finding is contrary to previous studies [11,21,46,47]. However, the concept of predicting behaviour by implicit measures was recently challenged by studies demonstrating that correlations between implicit measures and measures of behaviour are often small to medium[48–50]. Person-, context-, and behaviour-specific moderators might be responsible for these contradictory findings [25,48,49]. For instance, medication-taking behaviour can be seen as a more habitual, recurrent behaviour that might initially originate in conscious thought processes (“I should not forget my medication”), whereas some of the other examined behaviours can be argued to be less habitual and frequent (e.g., voting) and less rooted in conscious thought (e.g., brand preferences). The second explanation for the current findings might be that implicit processes do underlie objectively measured medication-taking behaviour, but were not detected in our study due to methodological limitations, described below.

### Methodological considerations

The key strengths of this study are the large sample size and patient recruitment in two of the largest rheumatology specialized centres across the Netherlands (i.e. covering approximately 20% of all patients with RA). Another strength is the use of MEMS to objectively measure medication-taking behaviour over three months in addition to the assessment of medication-taking behaviour by self-report. Measuring medication-taking behaviour by self-report is more susceptible to recall bias [30]. In contrast with Rüsch *et al*, we used validated questionnaires to assess self-reported adherence and beliefs about medicines [28]. An advantage of using both (validated) questionnaires and MEMS is the ability to compare both measurement instruments. However, MEMS is considered as gold standard method, since this method provides insight in more objectively measured daily medication-taking behaviour over time compared with self-report at one time point [51]. Still, it is an assumption that MEMS device usage corresponds with actual medication intake since intentional non-adherence to medication (e.g. opening MEMS without taking the medication) cannot be prevented in the home setting of patients. The awareness of being monitored (i.e. Hawthorne effect), might have contributed to the large proportion of adherent patients (i.e. the small amount of variation in adherence measures, in particular when MEMS are used) that we have found in our study when medication-taking behaviour was measured with both self-report and electronic drug monitors. This small amount of variation might have limited the power of this study to detect differences between adherent and non-adherent patients across attitudinal profiles, since high adherence rates could indicate more conscious and rational (e.g. planned) intake behaviour or high patient engagement with their treatment, in which case explicit attitudes and beliefs about medicines would override (potentially different) implicit associations. As a consequence, this might also limit the generalizability of the results.

The validity of the SC-IATs used in this study might be questioned since the target population might have a limited hand function, which might provide insufficient contrast between the experimental rounds in the SC-IATs. Also, it is unclear if the words and pictures used as stimuli in the SC-IATs are optimally related to patient’s medication use [49]. However, pictures were created based on pharmacy records in participating centres (i.e. manufacturer of the drugs, type of packaging, and appearance of the drug) and SC-IATs were personalised based on patient’s cDMARD use to increase the ability of patients to recognise their cDMARD at a glance. Another methodological consideration was the cut-off score for categorizing patients in attitudinal profiles based on (scale) midpoints for positive-negative attitudes and health-sickness related associations. The large proportion of patients who displayed relatively neutral implicit attitudes and associations (i.e. *D* measure near midpoint 0) or neutral explicit attitudes or health-related associations (i.e. mean score near midpoint 3) might have reduced the contrast in characteristics (e.g. patient-, clinical-,and treatment-related variables, beliefs about medicines, medication-taking behaviour, and clinical outcomes) across attitudinal profiles. Currently, there is discussion in the literature on whether implicit measures by definition expose automatic associations or subconscious attitudes or whether they might also be sensitive to more reflective and conscious thought [25,26,48]. There is no consensus yet on the terminology and nature of these implicit and explicit processes and measurements, on how these constructs interact with each other, and how stable these constructs are over time and across situations [23,25,26,48]. Future research should take these considerations into account.

### Generalizability of the results

The external validity of the results might be questionable due to the low response rate of patients, together with the high proportion of adherent participants. The latter might indicate selection bias since it is well known that in adherence research often only highly motivated patients are willing to participate in studies [52]. It is conceivable that our findings on MEMS adherence are, therefore, an overrepresentation of adherence rates in the general RA population. Also the large proportion of patients who had a Dutch ethnic background in a study site which is located in a multicultural setting, together with the long disease duration, support the idea of selection bias. Since we did not measure health literacy in this study, it is unknown if patients with low health literacy skills were underrepresented in our study sample. However, when comparing study participants to the general RA population in the Sint Maartenskliniek (chosen as reference due to limited access of data of non-participating patients across study sites), no significant differences were found in mean age, sex, and mean disease duration. The proportion of study participants using a biologic DMARD was smaller than the proportion of bDMARD users in the general population in the Sint Maartenskliniek (32.7% and 41.5% respectively), whereas the proportion of patients who are in remission was higher in the general RA population in the Sint Maartenskliniek compared to our study sample (71.5% and 41.7% respectively). The latter might also explain the large proportion of adherent patients, since patients with high disease activity scores might be more motivated to participate in this study and might also be more likely to adhere to their treatment during the study. It is also assumed that our findings on patient’s implicit and explicit attitudes towards cDMARDs cannot simply be extrapolated to biologic DMARDs and the recently introduced JAK-inhibitors. Taking together, our study population might not be optimally representative for the entire RA population, especially for patients with early RA, and ethnic minorities.

In conclusion, about half of the patients with RA showed incongruent (implicit and explicit) attitudes and health-related associations with cDMARDs. Implicit attitudes and associations had no additional value in explaining medication-taking behaviour and clinical outcomes over and above often used explicitly measured characteristics, attitudes and outcomes in the studied population. However, this research provides interesting areas for future research regarding implicit and explicit processes that might be involved in medication-taking behaviour.

## Supporting information

S1 File Design, procedures and analyses of SC-IATs. (PDF)

S2 File Bipolar Evaluative Adjective Scale. (PDF)

S1 Table List of abbreviations. (PDF)

S2 Table Attitudinal profiles: Health versus sickness associations with cDMARDs. Description of profiles of patients diagnosed with rheumatoid arthritis based on (in)congruent implicit and explicit health- versus sickness-related associations with cDMARDs. Categories were low versus medium-high for educational level, living alone versus living together (with children and/or partner) for residential status, and for ethnic background Dutch versus other. Variables with P-values ≤0.05 (unadjusted for multiple testing) were further analyzed with Bonferroni corrected post-hoc tests. Abbreviations: DAS28-CRP (Disease Activity Score based on 28 joints and C-Reactive Protein), DMARD (disease-modifying antirheumatic drug), bDMARD (biologic DMARD), MEMS (Medication Event Monitoring System).
(PDF)
