# Supplementary material for: Implicit and explicit attitudes towards disease-modifying antirheumatic drugs as possible target for improving medication adherence
Source: PLoS One. 2019 Aug 30;14(8):e0221290. doi: 10.1371/journal.pone.0221290 (PMC6716669; doi:10.1371/journal.pone.0221290)
Supplement: S1 File — (PDF) [file pone.0221290.s001.pdf]

S1 File. Design, procedures and analyses of SC-IATs

Detailed description of SC-IAT procedures

See Table 1 in S1 File for an example procedure of one of the four counterbalanced SC-IAT versions including two practice rounds and four experimental rounds. In the first practice round, trials consisted of a positive or a negative stimulus (i.e. pictures or words, such as pictures of a happy face or crying man, or the words ‘happy’ and ‘bad’), which patients had to categorise into a positive or negative category by using the keys ‘A’ (left key for positive categorisation) and ‘L’ (right key for negative categorisation) on the keyboard. Those categories were counterbalanced, so that half of the participants had to use the opposite keys for these categorisation tasks. During the first practice round a researcher was available to answer any questions. After this practice round, the researcher left the room to avoid unintentional distraction, which may affect the response times in the experimental rounds to measure implicit attitudes towards cDMARDs. In the two experimental rounds for attitudes towards cDMARDs, the category ‘drug’ was added to the categorisation task. In each round, besides 10 positive (i.e. five words and five pictures) and 10 negative stimuli (i.e. five words and five pictures), also 5 drug stimuli (i.e. pictures) were presented, matching patients personal cDMARD use (Table 2 in S1 File). Pictures representing positive stimuli included a laughing woman, thumbs up, a schematic yellow happy smiley, a cartoon of a shining sun and a red heart, whereas pictures representing negative stimuli included a crying man, thumbs down, a schematic yellow sad smiley, clouds with lightning, and a broken heart. If patients used more than one cDMARD, the following hierarchical sequence was followed to reduce the number of tests: 1) methotrexate, 2) leflunomide, 3) hydroxychloroquine, 4) sulfasalazine, 5) azathioprine. So, for example if a patient uses both methotrexate and hydroxychloroquine, the methotrexate version of the SC-IAT procedure was performed (see Table 2 in S1 File for the pictures used in the six SC-IAT versions). Procedures for the concept of health-related associations were identical to the procedures for attitudes towards cDMARDs described above, but positive-negative response categories and stimuli were replaced with health-sickness response categories and stimuli. Pictures representing health-related associations included a fit woman, a little girl eating healthy snacks, a running woman, a man with arms wide open, and hands in the shape of a heart on a belly, whereas pictures representing sickness-related associations included a little girl with a thermometer in her mouth, a sick woman, a man with hands on his head due to a headache, a woman with hands on her neck and a painful look, and two hands on a belly with a red (painful) appearance on the belly. Patient’s subject number and cDMARD use were decisive in assigning one of the four counterbalanced SC-IAT versions.

Round	Description	Trials	Left key (A)	Right key (L)
1	Practice round attitudes	20	Negative	Positive
2	Positive-negative attitudes	40	Negative + Medicine	Positive
3	Positive-negative attitudes	40	Negative	Positive + Medicine
4	Practice round health-related associations	20	Sickness	Health
5	Health-sickness associations	40	Sickness + Medicine	Health
6	Health-sickness associations	40	Sickness	Health + Medicine

S1 File Table 1. Example procedure of one of the four counterbalanced versions of the personalised Single Category Implicit Association Tests including two practice rounds and four experimental rounds.

Round	Stimulus 1	Stimulus 2	Stimulus 3	Stimulus 4	Stimulus 5
Stimuli MTX tablets	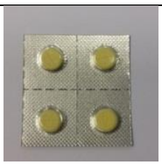	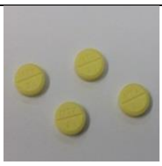	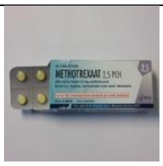	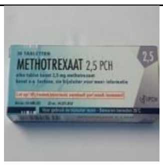	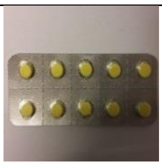
Stimuli MTX sub-cutaneous injections	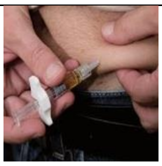	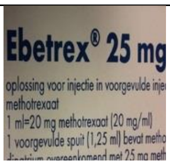	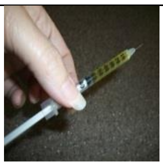	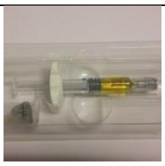	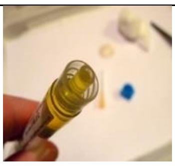
Stimuli leflunomide	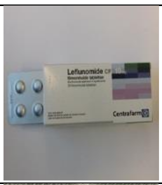	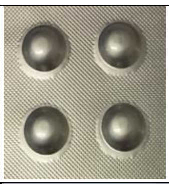	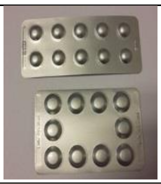	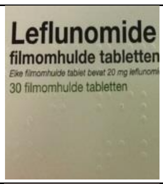	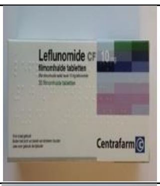
Stimuli hydroxyl-chloroquine	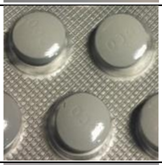	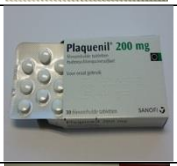	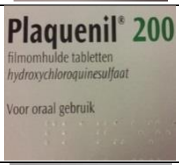	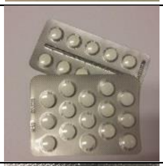	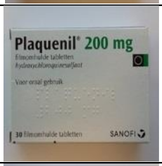
Stimuli azathioprine	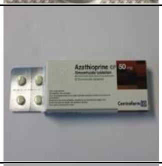	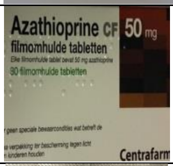	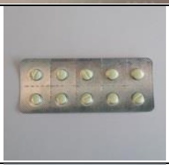	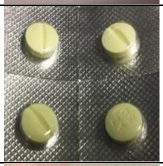	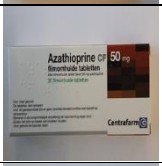
Stimuli sulfasalazine	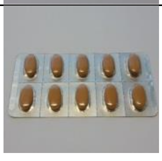	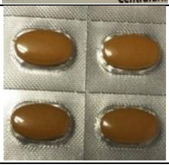	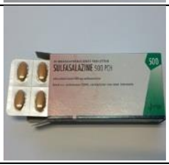	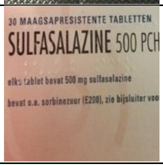	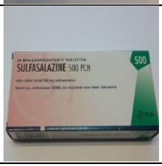

S1 File Table 2. Pictures/ drug stimuli used in each round of the different SC-IAT versions. Stimuli in the positive, negative, health and sickness round were used in all of the six personalised SC-IAT versions.

Statistical method to calculate *D* measure for the strength of automatic associations

The improved IAT scoring algorithm described by Greenwald *et al* was used to calculate the *D* measure for strength of automatic associations. According to this procedure, trials with latencies >10.000ms were excluded for data-analysis to avoid contamination of explicit attitudes or health-related associations; participants with latencies <300ms in more than 10% of all trials were excluded. Incorrect trials in the experimental rounds were replaced by the round's mean response time with a time penalty of +600ms. Next, differential scores were calculated on the two complementary SC-IAT rounds; the mean response time on the positive categorisation trials (drug-positive or drug-health) were subtracted from those on the negative categorisation trials (drug-negative or drug-sickness). As a result, larger values indicate relatively fast drug-positive (versus drug-negative) responses. Next, each differential score was divided by its associated pooled-trials SD. *D* measures above 0 indicate that patients had relatively faster response times on positive categorisation rounds than on negative categorisation rounds, and were interpreted as relatively positive (over negative) implicit attitudes towards cDMARDs or relatively stronger health- (than sickness) related associations, and vice versa.
