# Supplementary material for: Implicit and explicit attitudes towards disease-modifying antirheumatic drugs as possible target for improving medication adherence
Source: PLoS One. 2019 Aug 30;14(8):e0221290. doi: 10.1371/journal.pone.0221290 (PMC6716669; doi:10.1371/journal.pone.0221290)
Supplement: S2 File — (PDF) [file pone.0221290.s002.pdf]

S2 File. Bipolar evaluative adjective scale

We are interested in your opinion about the anti-rheumatic drug [name cDMARD] that we have just delivered.

For example: your opinion in general about your anti-rheumatic drug is not ‘tasteless’ nor ‘tasty’. You can tick one of the three boxes between the terms ‘Tasteless’ and ‘Tasty’. If you totally agree with one of the terms below, you can tick the box to the far left or right.

**My opinion about [name cDMARD] is:**						
Negative	□	□	□	□	□	Positive
Bad	□	□	□	□	□	Good
Redundant	□	□	□	□	□	Useful
Dangerous	□	□	□	□	□	Safely
Nasty	□	□	□	□	□	Pleasant
Tasteless	□	□	□	□	□	Tasty
Harmful	□	□	□	□	□	Curative
Sadness	□	□	□	□	□	Pleasure
Stupid	□	□	□	□	□	Nice
Ugly	□	□	□	□	□	Beautiful

**To what extent do you associate [name cDMARD] with the following terms:**						
Miserable	□	□	□	□	□	Excellent
Dead	□	□	□	□	□	Alive
Suffering	□	□	□	□	□	Enjoying
Drained	□	□	□	□	□	Energetic
Pain	□	□	□	□	□	Healing
Tired	□	□	□	□	□	Fresh
Limp	□	□	□	□	□	Powerful
Weak	□	□	□	□	□	Strong
