# Supplementary material for: Implicit and explicit attitudes towards disease-modifying antirheumatic drugs as possible target for improving medication adherence
Source: PLoS One. 2019 Aug 30;14(8):e0221290. doi: 10.1371/journal.pone.0221290 (PMC6716669; doi:10.1371/journal.pone.0221290)
Supplement: S1 Table — (PDF) [file pone.0221290.s003.pdf]

S1 Table. List of abbreviations

Abbreviation	Full name
Anti-CCP	Anti- cyclic citrullinated peptide
bDMARD	Biologic disease-modifying antirheumatic drug
BMQ-Specific	Beliefs about Medicines Questionnaire Specific
cDMARD	Conventional disease-modifying antirheumatic drug
CQR	Compliance Questionnaire on Rheumatology
DAS28-ESR	Disease Activity Score based on 28 joints and erythrocyte sedimentation rate
DAS-28 CRP	Disease Activity Score based on 28 joints and C-Reactive Protein
DMARD	Disease-modifying antirheumatic drug
EMERGE	ESPACOMP Medication Adherence Reporting Guideline
ESPACOMP	European Society for Patient Adherence and Compliance
IAT	Implicit Association Test
MEMS	Medication Event Monitoring System
NCD score	Necessity-concerns differential score
RA	Rheumatoid Arthritis
SC-IATS	Single Category Implicit Association Tests
STROBE	Strengthening the Reporting of Observational Studies in Epidemiology
