# Supplementary material for: Implicit and explicit attitudes towards disease-modifying antirheumatic drugs as possible target for improving medication adherence
Source: PLoS One. 2019 Aug 30;14(8):e0221290. doi: 10.1371/journal.pone.0221290 (PMC6716669; doi:10.1371/journal.pone.0221290)
Supplement: S2 Table — Description of profiles of patients diagnosed with rheumatoid arthritis based on (in)congruent implicit and explicit health- versus sickness-related associations with cDMARDs. Categories were low versus medium-high for educational level, living alone versus living together (with children and/or partner) for residential status, and for ethnic background Dutch versus other. Variables with P-values ≤0.05 (unadjusted for multiple testing) were further analyzed with Bonferroni corrected post-hoc tests. Abbreviations: DAS28-CRP (Disease Activity Score based on 28 joints and C-Reactive Protein), DMARD (disease-modifying antirheumatic drug), bDMARD (biologic DMARD), MEMS (Medication Event Monitoring System). (PDF) [file pone.0221290.s004.pdf]

S2 Table. Attitudinal profiles: health versus sickness associations with cDMARDs

S2 Table. Description of profiles of patients diagnosed with rheumatoid arthritis based on (in)congruent implicit and explicit health- versus sickness-related associations with cDMARDs

		Profile I	Profile II	Profile III	Profile IV	P-value
**Associations with cDMARDs**	**Explicit**	Health	Health	Sickness	Sickness	
	**Implicit**	Health (N=71)	Sickness (N=94)	Sickness (N=54)	Health (N=26)	
**Patient characteristics**						
Age in years, mean (SD)		63.6 (10.6)	63.4 (10.7)	59.6 (12.8)	62.9 (10.2)	0.38
Female, N (%)		53 (74.6)	59 (62.8)	36 (66.7)	17 (65.4)	0.44
High educational level, N (%)		25 (35.2)	34 (36.2)	14 (25.9)	8 (30.8)	0.60
Living alone, N (%)		20 (28.2)	18 (19.1)	9 (16.7)	7 (26.9)	0.35
**Clinical characteristics**						
Disease duration in years, mean (SD)		12.6 (9.7)	12.3 (8.7)	10.0 (9.1)	11.3 (9.0)	0.82
Anti-CCP positive, N (%)		47 (66.2)	59 (62.8)	36 (66.7)	19 (73.1)	0.94
Number of comorbidities, mean (SD)		2.2 (2.0)	2.2 (1.7)	2.2 (1.6)	1.6 (1.7)	0.43
**Treatment characteristics**						
Number of DMARDs, mean (SD)		1.5 (0.5)	1.5 (0.6)	1.8 (0.6)	1.5 (0.6)	**0.03**
Using bDMARDs, N (%)		23 (32.4)	24 (25.5)	26 (48.1)	7 (26.9)	**0.04**
**Beliefs about medicines**						
Necessity-concerns differential, mean (SD)		7.7 (4.8)	6.6 (4.4)	3.6 (5.0)	1.6 (5.2)	**<0.0001**
**Study outcomes**						
Correct dosing: proportion of adherent patients based on self-report, N (%)		69 (97.2)	86 (91.5)	50 (92.6)	20 (76.9)	**0.01**
Correct dosing, proportion of adherent		56 (78.9)	79 (84.0)	40 (74.1)	18 (69.2)	0.67

patients based on MEMS, N (%)					
DAS28-CRP, mean (SD)	2.0 (0.8)	2.2 (1.1)	2.6 (1.1)	2.6 (1.2)	0.08
Proportion of patients in remission, N (%)	35 (49.3)	38 (40.4)	18 (33.3)	10 (38.5)	0.40

Categories were low versus medium-high for educational level, living alone versus living together (with children and/or partner) for residential status, and for ethnic background Dutch versus other. Variables with P-values ≤0.05 (unadjusted for multiple testing) were further analyzed with Bonferroni corrected post-hoc tests. Abbreviations: DAS28-CRP (Disease Activity Score based on 28 joints and C-Reactive Protein), DMARD (disease-modifying antirheumatic drug), bDMARD (biologic DMARD), MEMS (Medication Event Monitoring System).
